# Associated degeneration of ventral tegmental area dopaminergic neurons in the rat nigrostriatal lactacystin model of parkinsonism and their neuroprotection by valproate

**DOI:** 10.1016/j.neulet.2015.12.052

**Published:** 2016-02-12

**Authors:** Ian F. Harrison, Hiba K. Anis, David T. Dexter

**Affiliations:** a UCL Centre for Advanced Biomedical Imaging, Division of Medicine, University College London, 72 Huntley Street, London WC1E 6DD, UK; b Parkinson’s Disease Research Group, Centre for Neuroinflammation and Neurodegeneration, Division of Brain Sciences, Department of Medicine, Imperial College London, Hammersmith Hospital Campus, London W12 0NN, UK

**Keywords:** PD, Parkinson’s disease, SNpc, Substantia Nigra pars compacta, αSyn, α-synuclein, VTA, ventral tegmental area, UPS, ubiquitin proteasome systems, HAT, histone acetyltransferase, HDAC, histone deacetylase, HDACI, histone deacetylase inhibitor, TH, tyrosine hydroxylase, Parkinson’s disease, Lactacystin, Ventral tegmental area, Substantia Nigra pars compacta, Dopamine, Histone deacetylase inhibitor, Valproate

## Abstract

Parkinson’s disease (PD) manifests clinically as bradykinesia, rigidity, and development of a resting tremor, primarily due to degeneration of dopaminergic nigrostriatal pathways in the brain. Intranigral administration of the irreversible ubiquitin proteasome system inhibitor, lactacystin, has been used extensively to model nigrostriatal degeneration in rats, and study the effects of candidate neuroprotective agents on the integrity of the dopaminergic nigrostriatal system. Recently however, adjacent extra-nigral brain regions such as the ventral tegmental area (VTA) have been noted to also become affected in this model, yet their integrity in studies of candidate neuroprotective agents in the model have largely been overlooked. Here we quantify the extent and distribution of dopaminergic degeneration in the VTA of rats intranigrally lesioned with lactacystin, and quantify the extent of VTA dopaminergic neuroprotection after systemic treatment with an epigenetic therapeutic agent, valproate, shown previously to protect dopaminergic SNpc neurons in this model. We found that unilateral intranigral administration of lactacystin resulted in a 53.81% and 31.72% interhemispheric loss of dopaminergic SNpc and VTA neurons, respectively. Daily systemic treatment of lactacystin lesioned rats with valproate however resulted in dose-dependant neuroprotection of VTA neurons. Our findings demonstrate that not only is the VTA also affected in the intranigral lactacystin rat model of PD, but that this extra-nigral brain region is substrate for neuroprotection by valproate, an agent shown previously to induce neuroprotection and neurorestoration of SNpc dopaminergic neurons in this model. Our results therefore suggest that valproate is a candidate for extra-nigral as well as intra-nigral neuroprotection.

## Highlights

• Intranigral lactacystin causes degeneration of adjacent VTA dopaminergic neurons.
• Valproate is protective to VTA neurons in the lactacystin rat model of Parkinson’s.
• Valproate is a candidate for extra-nigral as well as intra-nigral neuroprotection.

## 1 Introduction

Parkinson’s disease (PD) is a complex movement disorder classically characterised by clinical presentation of rigidity, tremor and bradykinesia, primarily resulting from degeneration of the dopaminergic nigrostriatal pathway [10]. Degenerating dopaminergic neurons within the Substantia Nigra pars compacta (SNpc) of PD patients display intracytoplasmic protein inclusions, known as Lewy bodies and Lewy neurites, composed predominantly of a misfolded synaptic protein called α-synuclein (αSyn) [30]. As PD develops this αSyn pathology is thought to progressively spread rostrally throughout the brain, gradually affecting higher order centres with disease progression, bringing about dementia and neuropsychiatric symptoms which are synonymous with late stage PD [2].

It has recently been suggested that the misfolding of αSyn is self-propagating, spreading from neuron to neuron *via* a ‘prion-like’ mechanism [16], [18]. The concept of spreading αSyn pathology in PD, particularly by direct transfer of αSyn between neurons suggests that there would be neuronal cell death in regions spatially nearby the SNpc, where αSyn pathology in PD is most evident. The SNpc lies just lateral to another dopaminergic centre, the ventral tegmental area (VTA), which like the SNpc has reciprocal projections to caudal nuclei, such as mesocortical projections to the prefrontal, orbitofrontal and cingulate cortices [23]. Spread of αSyn pathology in the brain and the proximity of the SNpc to the VTA would suggest that this region is also affected in PD. Correspondingly, it has previously been shown that this is the case in PD: VTA neuron numbers being reduced in PD brains compared to control [9], [28] thought, possibly to be related to duration or severity of depression in PD patients as these neurons normally function as part of the brain’s positive affect system, in reward or reinforcement processes [6].

Toxin based models of PD tend to focus exclusively on nigrostriatal neurodegeneration and although the SNpc and striatum are obvious regions of interest, such study designs do not consider the progressive nature of PD in which several neuronal systems gradually become affected. A recent animal model of PD takes advantage of the irreversible ubiquitin proteasome system (UPS) inhibitor, lactacystin, to model parkinsonian nigrostriatal neurodegeneration by causing intracytoplasmic accumulation of altered proteins [19], [20] and degeneration of dopaminergic and non-dopaminergic SNpc neurons [12]. Additionally however, when injected into the SNpc of rats, lactacystin has recently been observed to produce both intra- as well as extra-nigral pathology: affecting nearby regions such as the pedunculopontine nucleus (PPN) [26], the VTA [17] and also more rostral regions such as the primary motor (M1) and somatosensory cortices [32]. It remains unanswered however as to whether these neuronal populations susceptible to lactacystin induced cell death are also substrates for neuroprotective therapies known to successfully target nigrostriatal dopaminergic neurons in this model.

In healthy cells, histone acetylation is carefully controlled by the activity of two enzyme classes: histone acetyltransferases (HATs) which acetylate histone N-terminal tails, and histone deacetylases (HDACs) which deacetylase histone N-terminal tails. It was discovered recently that αSyn ‘masks’ histone proteins, preventing their acetylation, and as such the resulting histone hypoacetylation is thought to contribute to neurodegeneration in PD [14]. In addition it has been observed that a misbalance in the activities of HATs and HDACs exists in neurodegenerative scenarios, leading to an exacerbation of the histone hypoacetylation and associated apoptosis [29]. We therefore postulate that this deregulation of the balance of histone acetylation and deacetylation could be rectified with the use of HDAC inhibitors (HDACIs), to reduce the neurodegeneration observed as a result of histone hypoacetylation in PD [8]. Correspondingly, we have shown recently that systemic treatment with valproate, an inhibitor of HDAC classes I (HDACs 1–3 & 8) and IIa (HDACs 4, 5, 7 & 9), causes neuroprotection and neurorestoration of dopaminergic neurons within the SNpc of lactacystin lesioned rats [7]. The SNpc is known to express HDACs 2–5 and 11 most abundantly, as does the VTA [3]. Therefore, if valproate is to cause protection/restoration of neurons in the SNpc by inhibition of HDACs it would therefore be likely that valproate also protects against lactacystin induced neurodegeneration in the VTA.

In this study, we firstly aim to quantify the effect of a nigral proteasome inhibitor lesion on the integrity of the nearby VTA in the rat brain, with specific focus on the extent and distribution of dopaminergic neuronal toxicity within this region. Secondly we seek to determine whether or not dopaminergic VTA neurons, like SNpc neurons, are a substrate for valproate mediated neuroprotection/restoration in this proteasome inhibitor model of PD.

## 2 Materials and methods

### 2.1 Experimental animals

Animal procedures were carried out in accordance with the Home Office Animals (Scientific Procedures) Act, UK, 1986 and were previously approved by the Imperial College Animal Welfare and Ethical Review Board. Male Sprague-Dawley rats (260 ± 10 g, Charles River, UK) were housed in groups of two or three at 21 ± 1 °C on a 12 h light–dark cycle with the relative humidity maintained at 55 ± 10%. Standard rat chow and drinking water were available *ad libitum* throughout the duration of the study, and were supplemented with standard rat wet diet for seven days post-surgery.

### 2.2 Animal treatment groups

**Fig. 1:**
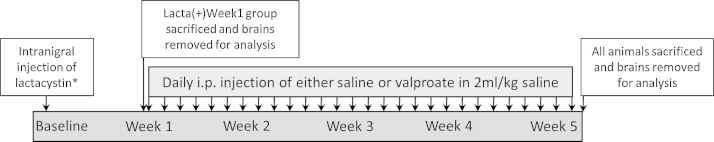
Animal study design. *Lacta(+) Week 1, Lacta(+)VPA(−), Lacta(+)VPA(+) and Lacta(+)VPA(++) groups were intranigrally injected with lactacystin. Control groups (Lacta(−)VPA(−) and Lacta(−)VPA(++)) remained surgically naïve.

Six animal treatment groups were included in the study (Table 1): four groups which received intranigral injection of lactacystin and were either culled 7 days post-surgery, or subsequently treated for 28 days with either saline or valproate (200 or 400 mg/kg) administered i.p. starting 7 days post-surgery, and two groups which remained surgically naïve whilst receiving subsequent treatment with either saline or valproate (400 mg/kg). See Fig. 1 for study design.

### 2.3 Stereotaxic lesioning of the SNpc with lactacystin

The left SNpc was stereotaxically lesioned using the irreversible proteasome inhibitor lactacystin (Enzo Life Sciences, Exeter, UK) as previously described by us [7], [26] and others [5], [22], [31], [32], [33]. Briefly, isoflurane (IsoFlo^®^, Abbot Laboratories, Maidenhead, UK) anaesthetised animals were positioned in a stereotaxic frame (Kopf Instruments, Tujunga, USA) in the horizontal skull position, and a midline incision made in the scalp to expose the skull, where bregma was identified [24]. A small burr hole was made in the skull above the SNpc and 10 μg of lactacystin (2.5 μg/μl in sterile (0.9%) saline, 4 μl in total) was stereotaxically administered *via* a Hamilton syringe to the SNpc: anterio-posterior, −5.2 mm, medio-lateral, +2.5 mm and ventral to dura, −7.6 mm [24]. The lactacystin was injected at a rate of 1 μl/min, the needle was left *in situ* for 3 min before being retracted. The wound was sutured closed and animals left to recover in a heated recovery chamber before being returned to their home cages.

### 2.4 Tissue collection and preparation

At the end of the study period, animals were sacrificed, decapitated and the brain removed from the skull. Brains were fixed in 4% paraformaldehyde in phosphate buffered saline (PBS) (pH 7.4) for 72 h before being cryoprotected in 30% sucrose in PBS until the tissue was observed to have sunk. Blocks were then snap frozen in isopentane pre-chilled on dry ice and stored at −80 °C for subsequent sectioning. 30 μm thick coronal sections were collected throughout the extent of the SNpc and VTA onto SuperFrost^®^ Plus slides (VWR international, Lutterworth, UK) using a cryostat (Bright Instruments, Huntingdon, UK). Slides were then stored at −80 °C until immunohistochemical staining.

### 2.5 Immunohistochemistry

Immunohistochemistry for tyrosine hydroxide (TH), the rate limiting enzyme in monoamine synthesis was utilised as the cellular marker for dopaminergic neurons in the SNpc and VTA, and cresyl violet was used as a counterstain for the Nissl body of all neurons. For this the avidin–biotin complex (ABC)/peroxidase method of immunohistochemistry with a cresyl violet counterstain was performed similar to that previously published by our group [7]. Briefly, endogenous peroxidase activity and non-specific binding were blocked with H_2_O_2_ and normal goat serum respectively, prior to incubation of tissue sections with the primary antibody (Rabbit Polyclonal Anti-Tyrosine Hydroxylase, Millipore, MA, USA) at 1:1000. Sections were then incubated with the secondary antibody (Biotinylated Goat Anti-Rabbit Secondary Antibody, VectorLabs, Peterborough, UK) at 1:200 before being washed an incubated with ABC (Vectastain Elite ABC Kit, VectorLabs). Staining was then visualised with 3,3′-diaminobenzidine (DAB) prior to being counterstained with cresyl violet, dehydrated and coverslipped.

### 2.6 Quantification of SNpc and VTA neurons

Images of each section containing either the SNpc or VTA were acquired using ImagePro7 software (MediaCybernetics Inc., Bethesda, MD, USA) and captured using a Nikon Eclipse E1000 M microscope/digital camera system (Nikon Instruments, Surrey, UK) at 10× magnification. The SNpc and VTA were both delineated manually referring to previously published morphological boundaries and a rat brain atlas [4], [24] to create an area of interest (AOI). Counts of both the TH positive (TH+) and Nissl positive (Nissl+) cells were made of the entire AOI in both hemsipheres using ImageJ (v1.4, National Institutes of Health, Bethesda, MD, USA). An adaptation of the optical fractionator method of stereological cell number estimation was utilised in accordance with previously published protocols [34]. For this, every 6th section in the series containing the AOI was analysed giving a section sampling fraction (ssf) of 1/6. A Heidenhain microcator (Hedenhain, Traunreut, Germany) attached to a Nikon Eclipse E800 microscope (Nikon Instruments) with a JVC 3CCD camera (JVC, London, UK) was used to determine the height sampling fraction (hsf): measuring the section height of three random points within each AOI to give a mean section thickness. For calculation of the total cell estimates (*N*), the following equation was used, where n is the number of cells manually counted [34]:

$$N=n\left(\frac{1}{\text{hsf}}\right)\left(\frac{1}{\text{ssf}}\right)$$

### 2.7 Statistical analysis

Paired *t*-tests allowed comparison of SNpc and VTA cell loss in lactacystin lesioned animals. A two way ANOVA with Bonferroni post-test was used to compare cell numbers between hemispheres at each level of the VTA. Paired *t*-tests allowed comparison of cell counts in the ipsilateral and contralateral hemispheres for both cell types. A one way ANOVA with Bonferroni post-test was used to compare cell loss percentages between experimental groups. All data is presented as mean ± standard error of mean. All statistical tests were performed using GraphPad Prism (v5.0 for Windows, GraphPad Software, San Diego, CA, USA).

## 3 Results

### 3.1 Extent and distribution of TH+ and Nissl+ cell loss in the VTA of intranigrally lesioned rats

**Fig. 2:**
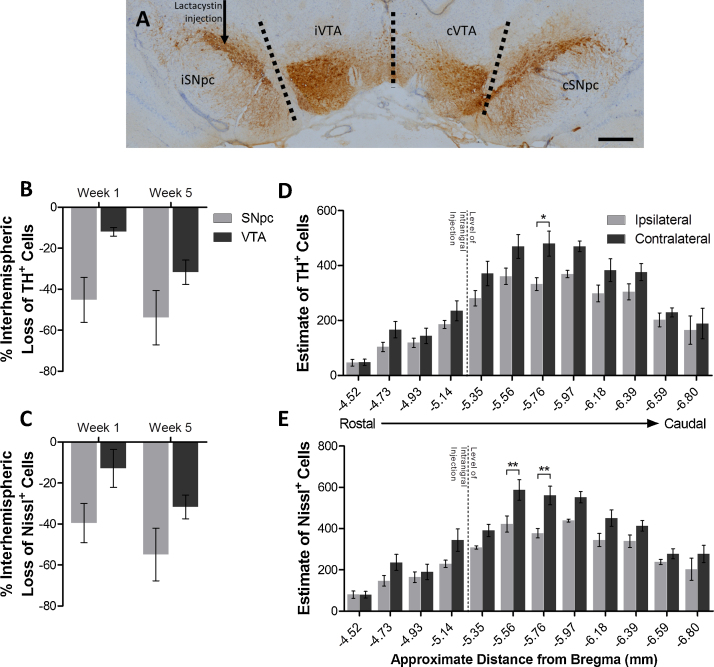
Lactacystin induced loss of TH+ and Nissl+ cell loss in the VTA. (A) Representative image of TH immunohistochemistry within the brain showing proximity and discrimination of the SNpc from the VTA. Mean percentage interhemispheric (ipsilateral lesioned *vs.* contralateral non-lesioned hemisphere) loss of (B) TH+ cells and (C) Nissl+ cells in the SNpc and VTA of lactacystin (10 μg) lesioned animals, 1 and 5 weeks post-lesion. Distribution of (D) TH+ and (E) Nissl+ cell loss at each rostrocaudal level of the VTA in intranigral lactacystin lesioned animals, 5 weeks post-lesion. Each level is defined by its approximate distance caudal to bregma. Dotted line represents the level of the intranigral injection of lactacystin. Data presented as mean ± SEM. Statistical significance indicated with asterisks: *p < 0.05, **p < 0.01. *n* = 6/7 per group. Scale bar equal to 500 μm. *Abbreviations*: iSNpc, ipsilateral Substantia Nigra pars compacta; cSNpc, contralateral Substantia Nigra pars compacta; iVTA, ipsilateral ventral tegmental area; cVTA, contralateral central ventral tegmental area.

As expected, intranigral lesioning with lactacystin resulted in a marked interhemispheric loss of TH+ and Nissl+ positive cells in the SNpc, observed 7 days post-lesion (Fig. 2B and C, % interhemispheric loss in the SNpc of TH+ and Nissl+ cells, week 1, 45.17 ± 10.95% and 39.50 ± 10.47%, respectively). This interhemispheric loss in the SNpc was modestly exacerbated, 5 weeks post-lesion (TH+ and Nissl+ cells loss, 1.19 and 1.39 fold increase from week 1). The VTA however, exhibited only a subtle interhemispheric loss of dopaminergic neurons, 7 days post-lesion, (Fig. 2B and C, % interhemispheric loss in the VTA of TH+ and Nissl+ cells, week 1, 11.94 ± 2.12% and 12.83 ± 9.24%, respectively) which was then markedly exacerbated 5 weeks post-lesion (TH+ and Nissl+ cells loss, 2.66 and 2.47 fold increase from week 1). However this loss in the VTA at week 5 was far less than that observed in the SNpc (Fig. 2B and C, % interhemispheric loss in the VTA of TH+ and Nissl+ cells, 22.10 and 23.25% less than that in the SNpc, respectively).

At week 5, in all caudorostral levels of the VTA examined, fewer TH+ and Nissl+ cells were observed in the lesioned hemisphere compared to the unlesioned hemisphere (Fig. 2D and E). However this interhemispheric loss of TH+ cells in the VTA was most evident in the centre of the nucleus where numbers of TH+ cells are greatest (TH+ VTA cells at −5.76 mm from bregma, ipsilateral, 332 ± 21 *vs.* contralateral, 480 ± 41 cells, 27.95 ± 4.86% interhemispheric loss, *p* < 0.05). Similar was the case with interhemispheric loss of Nissl+ cells: greatest loss being observed at −5.56 and −5.76 mm from bregma (27.56 ± 4.70% and 31.27 ± 2.23% interhemispheric loss of Nissl+ cells observed at −5.56 and −5.76 mm from bregma respectively, *p* < 0.01 in both comparisons).

### 3.2 Valproate mediated neurorestoration of TH+ and Nissl+ VTA neurons

**Fig. 3:**
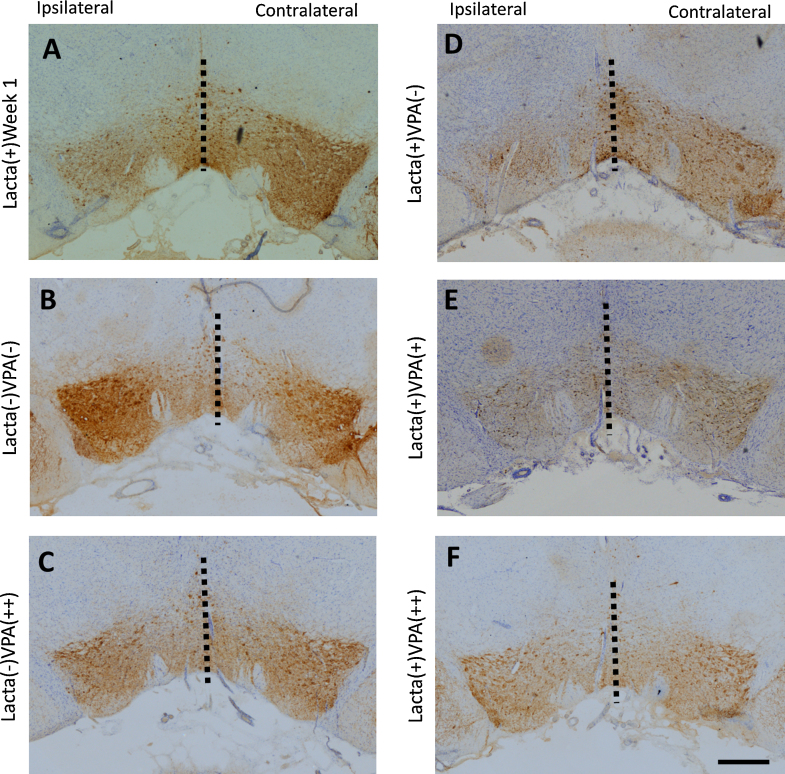
Micrographs demonstrating loss and protection of TH+ cells in the VTA. Representative example images of the TH and Nissl stained VTA in each treatment group: (A) Lacta(+) Week1; (B) Lacta(−)VPA(−); (C) Lacta(−)VPA(++); (D) Lacta(+)VPA(−); (E) Lacta(+)VPA(+); (F) Lacta(+)VPA(++). All sections are approximately −5.8 mm caudal to bregma. Scale bar equal to 500 μm.

**Fig. 4:**
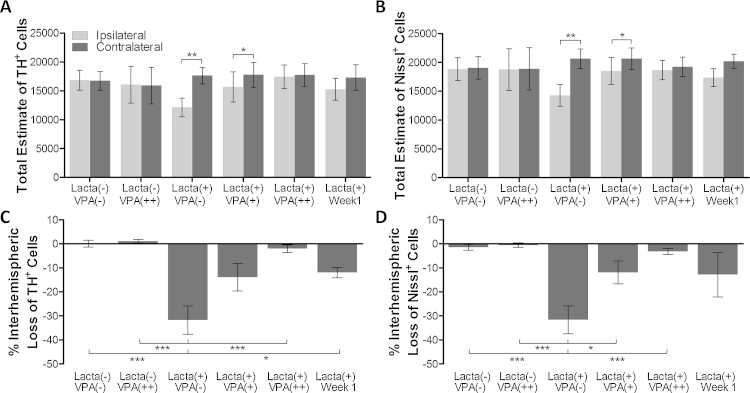
Valproate treatment causes dose-dependant protection of dopaminergic neurons in the VTA in lactacystin lesioned animals. Stereological estimated (A) TH+ and (B) Nissl+ neuron numbers in the VTA of rats suggest a dose-dependant neuroprotective/restorative effect of valproate in the intranigral lactacystin (10 μg) rat model of Parkinson’s disease. This is exemplified by the percentage interhemispheric loss of TH+ (C) and Nissl+ (D) neurons calculated between hemispheres of the VTA. Data presented as mean ± SEM. Statistical significance indicated with asterisks: *p < 0.05; **p < 0.01, ***p < 0.001. *n* = 6/7 per group.

No interhemispheric cell loss in the VTA was observed in either surgically naïve saline treated, or surgically naïve valproate treated, control animal groups (Fig. 3, Fig. 4, mean total of TH+ cells in the VTA, 32,838 ± 3624). These numbers compared favourably to those previously published [21]. As noted above, in animals intranigrally lesioned with lactacystin, and treated for 28 days with saline, there was a significant interhemispheric loss of both TH+ and Nissl+ cells in the VTA (Fig. 4, 31.71 ± 5.94 and 31.63 ± 5.79% interhemispheric loss, respectively). Animals lesioned with lactacystin which received subsequent treatment with valproate however displayed dose dependant protection of both TH+ and Nissl+ neurons in the VTA (Fig. 4C and D, interhemispheric loss of TH+ cells, 13.87 ± 5.73% and 2.03 ± 1.56% loss in 200 mg/kg and 400 mg/kg valproate treated animals respectively). This interhemispheric loss of TH+ neurons in animals treated with 400 mg/kg valproate reached significance from that in saline treated (*p *< 0.001). Similarly, interhemispheric loss of Nissl+ neurons was significantly greater in saline treated animal compared to both 200 mg/kg valproate treated animals (*p* < 0.05) and 400 mg/kg valproate treated animals (*p *< 0.001).

## 4 Discussion

We have shown here that intranigral injection of the irreversible proteasome inhibitor, lactacystin, in rats results in not only a significant degree of dopaminergic cell loss in the SNpc but also in the adjacent VTA. This loss of neurons in the VTA in this model is sustained throughout the entirety of the nucleus: the largest degree of cell loss appearing in the centre of the structure where cell numbers are greatest. Like the dopaminergic neurons of the SNpc, as we have previously published [7], we also show here that the lactacystin induced degeneration of dopaminergic neurons in the VTA is also dose dependantly ablated upon systemic treatment with the HDACI, valproate, suggesting that valproate is a candidate for both intra- and extra-nigral neuroprotection. Importantly, to our knowledge, this is the first study on the impact of a candidate neuroprotective agent on the integrity of the VTA in this animal model of PD: all previously published studies justifiably focussing on dopaminergic nigrostriatal integrity.

The irreversible proteasome inhibitor, lactacystin, has been shown extensively to recapitulate parkinsonian dopaminergic neuronal cell death, when stereotaxically injected into the nigrostriatal system of rats [15], [19], [22], [26], [31], resulting in formation of ubiquitin/αSyn immunopositive inclusions in nigral dopaminergic neurons [15], [19], [22], [26], [31]. This intracellular accumulation of altered proteins results in progressive nigral neurodegeneration and subsequent progressive development of motor behavioural deficits [31], [32]. These behavioural symptoms have also been observed to be attenuated with chronic l-DOPA treatment [13]. Therefore, unlike many other animal models of PD, the lactacystin model not only recapitulates the formation of protein inclusions in dopaminergic neurons, but also models the progressive nature of both dopaminergic neurodegeneration and development of l-DOPA attenuated motor behavioural deficits. These qualities make the lactacystin rat model of PD and ideal platform on which to study the neuroprotective potential of candidate therapeutics, allowing for simultaneous study of motor behaviour, neuropathology, and cellular and molecular changes in a single animal model.

As the lactacystin model becomes increasingly used in the field, more recently, focus has turned to the effects of intranigral lactacystin on the integrity of adjacent neuronal populations [12], [17], [26], [32]. The PPN, which lies just rostral to the SNpc, contains a mixed neuronal population of glutamatergic, γ-aminobutyric acid (GABA)-ergic and cholinergic neurons [27]. We showed recently that rats intranigrally injected with lactacystin displayed a marked loss of cholinergic neurons in the PPN [26]. Additionally, in this animal model Mackey et al. [17] have also studied the integrity of the VTA, which lies just lateral to the SNpc, demonstrating increased degeneration of dopaminergic VTA neurons with increasing dose of intranigral lactacystin. Importantly, consistent with their findings, we show here that the loss of VTA dopaminergic neurons in the model is less severe than in the SNpc. This relative vulnerability to UPS inhibition of the dopamine producing cells in the SNpc compared to those in the VTA is also consistent with post-mortem findings in human PD in which αSyn pathology affects dopaminergic neurons earlier in the course of disease than the VTA [2]. A number of differences exist between the dopaminergic neurons of the SNpc and those of the VTA, which could attribute to this relative vulnerability observed clinically and in this rodent model of PD. Firstly, dopaminergic neurons within the SNpc have typically 2–5 dendrites emanating from the poles of the neuron [1], [11], whereas dopaminergic neurons of the VTA have 3–5 dendrites emanating from the soma that extend in a radial array from the soma, probably due to the lack of spatial constraints in the VTA [1], [11]. Electrophysiologically, neither class of neuron differ in their tonic and burst firing rates, however VTA neurons exhibit a lower amplitude of slow oscillation in firing rate, compared to SNpc neurons, perhaps attributing to their reduced vulnerability in PD.

In the current study, degeneration was observed only in the ipsilateral VTA. However in contrast to this Mackey et al. [17] have previously demonstrated bilateral VTA degeneration in animals unilaterally injected with 10 μg lactacystin. Similarly, they observe a far greater degree of both ipsilateral VTA and SNpc degeneration at this dose. A number of differences exist between the study by Mackey et al., and ours however, which could attribute to the discrepancy in findings. The volume and concentration of the lactacystin containing solution was identical between studies however the coordinates used for intranigral injection were different (current study: AP −5.2 mm, ML +2.5 mm, VD −7.6; Mackey et al. [17]: AP −5.5 mm, ML −2 mm, DV −7.5). Of note to the VTA, the medio-lateral coordinate previously used by Mackey et al., is far closer (0.5 mm) to the midline, and it is therefore possible that injection of lactacystin at this location could attribute for the increased susceptibility of both the ipsilateral, and contralateral VTA to degeneration. Likewise, the stereotaxic brain atlas used as reference for these coordinates is noted only to be used for animals weighing between 250 and 350 g [24]. The animals used in the study by Mackey et al., are far larger than this (350–550 g), and given the known inaccuracy of brain stereotaxic coordinates when applied to animals outside of this weight range [25], it suggests that this may account for some of the differences in findings between studies of this intranigral dose of lactacystin. Lastly, the extent of VTA degeneration in the study by Mackey et al., was quantified 8–10 weeks after lactacystin lesioning, as opposed to 5 weeks in the current study. Lactacystin is an irreversible UPS inhibitor and therefore it is thought that its neurodegenerative affect would continue, again possibly explaining the discrepancy between our, and Mackey et al., studies.

We recently suggested that either transport or diffusion of the injected lactacystin may be responsible for toxicity in the nearby PPN, due to our observations of greater cell loss and neuronal atrophy in the rostral (side closest to the SNpc) compared to the caudal PPN [26]. In the current study however degeneration in the VTA was observed throughout the entirety of the structure: not focussed to the anterio-posterior level closest to the SNpc injection but to the centre of the structure where cell numbers are greatest. Moreover in the current study, lactacystin injection into the SNpc was conducted at a very low infusion rate, of just 1 μl/min, with the aim of limiting the free diffusion and mislocalisation of the toxin from site of injection. Similarly, post-injection, the needle was left *in situ* for three minutes, with the aim of limiting efflux of the toxin from the needle tract upon needle retraction and ensuring complete lactacystin absorption into the SNpc. This therefore suggests that the previous postulation of either transport or diffusion of lactacystin to nearby nuclei, affecting their integrity, is not responsible for intranigral lactacystin induced degeneration in the VTA observed here suggesting that mechanisms other than transport or diffusion of the toxin to nearby nuclei are responsible for spreading pathology. Further work is therefore required in order to understand how the spread of pathology observed in the current study is achieved.

Intracellular αSyn accumulation has been observed to actively promote histone hypoacetylation both *in vitro* in SH-SY5Y cells and *in vivo* in drosophila, both overexpressing αSyn [14]. This is thought to be achieved through αSyn ‘masking’ histone proteins, preventing their acetylation by HATs [14]. Notably we showed recently, that in line with its effects on αSyn accumulation *in vivo*, intranigral injection of lactacystin caused a significant reduction in histone acetylation in the brain [7]. Furthermore, this was able to be dose dependantly reversed upon treatment with the HDACI, valproate, translating to upregulation of neurotrophic and neuroprotective factors, and neuroprotection and restoration of degenerating dopaminergic neurons in the lesioned SNpc [7]. In these same animals, in the current study we demonstrate that dopaminergic neurons in the VTA too are subject to neuroprotection by treatment of the animals with valproate. The SNpc itself expresses HDACs 2–5 and 11 most abundantly [3], the majority of which are inhibited by valproate, an inhibitor of HDAC classes I (HDACs 1–3 & 8) and IIa (HDACs 4, 5, 7 & 9). The VTA expresses a similar profile of HDAC isoform expression to the SNpc, suggesting that if neuroprotection can be achieved through valproate’s inhibition of classes I and IIa in the SNpc, then the neuroprotection observed in the VTA may also be achieved *via* this mechanism. However, further analysis of the HDAC isoform expression profile of VTA dopaminergic neurons, and this cell type’s response to valproate treatment in terms of its histone acetylation status, needs to be performed in order to unambiguously suggest that they are subject to neuroprotection by HDAC inhibition. Likewise, valproate is a somewhat promiscuous drug: affecting GSK-3 and Akt/ERK pathways, GABA/glutamate neurotransmission, Na^+^ and Ca^2+^ voltage-dependant channels, phosphoinositol/TCA pathways and the oxidative phosphorylation pathway [35]. A number of these effects too could contribute towards valproate’s neuroprotective phenotype observed here and hence their involvement in this experimental setting should also be investigated in order to rule them out of the neuroprotective phenotype observed here.

## 5 Conclusions

We show here that intranigral injection of the irreversible proteasome inhibitor, lactacystin, causes not only neurodegeneration within the SNpc itself, but also extensively within the nearby VTA, 5 weeks post-lesion. The extent of degeneration observed in both of these regions demonstrate that this animal models well the extent of degeneration observed in the SNpc and VTA in human PD. Additionally, we demonstrate here that like neurons of the SNpc, degenerating dopaminergic neurons within the VTA are also a substrate for neuroprotection by the HDACI, valproate. These findings, along with those previously published; suggest that the lactacystin rat model of PD is an ideal model for the study of extranigral degeneration in PD with particular relevance for the study of the efficacy of neuroprotective drugs, designed initially for protection of SNpc neurons, on extranigral structures.

## Authorship contributions

I.F.H. contributed to the design of the study, performed experiments, and wrote the manuscript. H.K.A. performed experiments and analysis, and contributed to the manuscript critique. D.T.D. conceived and helped design the study, and contributed to the manuscript critique.

## Figures and Tables

**Table 1:** Animal treatment groups.

Group	*N=*	Intranigral injection	Daily i.p. injections^a^
Lacta(−)VPA(−)	7	None	Saline
Lacta(−)VPA(++)	6	None	Valproate (400 mg/kg)
Lacta(+)VPA(−)	7	Lactacystin (10 μg in 4 μl saline)	Saline
Lacta(+)VPA(+)	6	Lactacystin (10 μg in 4 μl saline)	Valproate (200 mg/kg)
Lacta(+)VPA(++)	6	Lactacystin (10 μg in 4 μl saline)	Valproate (400 mg/kg)
Lacta(+) Week1	6	Lactacystin (10 μg in 4 μl saline)	None (culled 1 week post-lesion)

a All daily i.p. injections given as 2 ml/kg: saline injections given as 2 ml/kg empty saline; 400 mg/kg valproate injections given as 2 ml/kg of 200 mg/ml solution of valproate in saline; 200 mg/kg valproate injections given as 2 ml/kg of 100 mg/ml solution of valproate in saline.
